# Outcomes of the Transsphenoidal Approach for ACTH-Secreting Pituitary Tumours and the Role of Postoperative ACTH in Predicting the Late Recurrence of Cushing’s Disease: A Retrospective Analysis of 50 Cases

**DOI:** 10.3390/healthcare13121395

**Published:** 2025-06-11

**Authors:** Athanasios Saratziotis, Maria Baldovin, Claudia Zanotti, Sara Munari, Luca Denaro, Jiannis Hajiioannou, Enzo Emanuelli

**Affiliations:** 1 Department of Otolaryngology, University Hospital of Larissa, 41334 Larisa, Greece; jhajiioannou@gmail.com; 2 Otorhinolaryngology Unit, San Martino Hospital, Belluno, ULSS1 Dolomiti, 32100 Belluno, Italy; maria.baldovin@gmail.com; 3 Otolaryngology Unit, S. Valentino Hospital, Montebelluna (TV), AULSS2 Marca Trevigiana, 31100 Treviso, Italy; zanotti.claudia89@gmail.com (C.Z.); saramunari@hotmail.it (S.M.); 4 Department of Neurosciences, Neurosurgery Section, University of Padua, 35122 Padova, Italy; luca.denaro@unipd.it; 5 Otolaringology Unit, Ca’ Foncello Hospital, Local Health Unit N.2 “Marca Trevigiana”, 31100 Treviso, Italy; enzoemanuelli@libero.it

**Keywords:** Cushing’s disease, ACTH-secreting pituitary adenoma, endoscopic transsphenoidal surgery, treatment outcome, postoperative cortisol, remission outcome

## Abstract

**Background/Objectives**: The endoscopic transsphenoidal approach constitutes an excellent technique for adrenocorticotropin hormone (ACTH)-producing pituitary tumours. It is associated with subnormal postoperative serum cortisol levels, which may guide decisions regarding immediate re-operation. **Methods**: The authors retrospectively reviewed patients with Cushing’s disease who had undergone endoscopic transsphenoidal surgery between 2013 and 2023. All operations were performed by neurosurgeons and skull-base otolaryngologists. Surgical outcomes were evaluated in combination with prognostic factors such as cortisol and ACTH levels in terms of long-term remission and late recurrence rates of Cushing’s disease. **Results**: Fifty patients aged between 15 and 69 (average 37.8) years were evaluated, having undergone 50 operations. The median follow-up was 76.5 months (range: 23–122 months). Major complications with a transient CSF leak resulting from the surgical approach occurred in three patients. Two patients in the series experienced minor complications, developing a deep vein thrombosis, and thirteen patients developed transient diabetes insipidus. The initial remission rate was 84% (n = 42/50). Initial non-remission occurred in eight (8) patients (16%), with three macro- and five microadenomas. A total of 3 of the 42 patients with initial remission had a late recurrence after 50 months follow-up and required repeat transsphenoidal surgery. Seven patients (16.6%) who did not exhibit early postoperative cortisol reduction subsequently achieved remission. Male gender was the only factor that was significantly associated with lower remission rates in either short- or long-term follow-up (*p* = 0.003 and 0.038, respectively). An immediate postoperative ACTH nadir of ≤5 pg/mL was significantly related to long-term remission (*p* = 0.004). In our study, a significant correlation was confirmed between remission of the disease and 24 h urinary cortisol values, both early and late (*p* = 0.019), and serum cortisol <138 nmol/L. In this retrospective study from a single institution specialising in pituitary tumour management, the endoscopic transsphenoidal approach was shown to be both safe and effective. Additionally, we found that the risk of relapse in patients with Cushing’s disease persisting for more than 5 years after surgery is real but low. Moreover, failure to achieve an early postoperative cortisol reduction does not preclude a subsequent remission. **Conclusions**: Our findings demonstrate that ACTH, postoperative serum cortisol, and urinary free cortisol are valuable predictors of relapse over a five-year period and are closely correlated to each other.

## 1. Introduction

Adrenocorticotropic hormone (ACTH)-secreting pituitary adenomas, causing Cushing’s disease (CD), have been found to be causal factors for persistent hypercortisolism, which is preferably treated with transsphenoidal surgery (TSS), and have been observed to be associated with immunosuppression, diabetes, hypertension, osteoporosis, and higher mortality rates. Upon the application of TSS, remission rates for patients with CD range from 41% to 98% [1]. The intended outcome of this surgery is increased long-term survival. Complete surgical resection of the pituitary adenoma results in longstanding corticotroph suppression in the normal gland. The effects observed postoperatively are hypocortisolism and a response of corticotropin-releasing hormone (CRH) suppression [2].

In our study, standardised protocols were followed before surgery. An MRI evaluation of the pituitary gland was carried out for all patients, including a triaxial contrast-enhanced MRI of the sella and parasellar regions, with dynamic contrast sequences. In order to confirm a central source of ACTH, a different approach of bilateral inferior petrosal sinus sampling (BIPSS) with CRH stimulation was adopted for some patients who had previously undergone surgery that was confirmatory for ACTH-producing tumours, and in patients where the adenoma was not visible on imaging. After a consensus diagnosis of CD was made by a multidisciplinary pituitary team, patients were evaluated for their suitability for TSS. A series of tests were performed, which included multiple measurements of 24 h urinary free cortisol (UFC) levels, and random plasma ACTH and cortisol levels, which included both elevated; and non-suppressible plasma cortisol levels (<5 mg/dL) with low-dose dexamethasone. Additionally, levels of midnight serum cortisol were found to be elevated and/or the disruption of salivary cortisol was detected, a characteristic of the diurnal adrenocortical secretion pattern.

The monitoring of remission from CD is carried out with low postoperative cortisol and ACTH levels serving as markers. It is worth noting, though, that the confirmation of early remission is not as simple as might be expected; there is some variation between publications in terms of cut-off levels, as well as sampling times [3,4]. This variability is believed to be due to differences in tumour size, cavernous sinus invasion, the presence of an ectopic tumour, perioperative glucocorticoid use, corticosteroid-binding protein (transcortin) level, and the extent of tumour resection. Postoperative basal cortisol levels also act as a long-term predictor of non-remission [5].

This means that new measurement methods must be devised. Variability in early postoperative cortisol and ACTH levels needs to be stabilised, and the accuracy of prediction of early non-remission needs to be improved. In the case of CD, the early detection of non-remission can benefit the patient, resulting in early TSS re-operation. Serum cortisol values provide a slope that begins immediately, at the onset of the postoperative period. In ACTH-secreting pituitary adenomas, because of the need to predict the completeness of tumour resection, this decline could serve as a biomarker. It would therefore be possible for patients to become eligible for enhanced biochemical follow-up, which may prevent a relapse of the disease [6].

At the time of diagnosis, most ACTH-secreting corticotroph adenomas are microadenomas, and in some cases, they may be difficult to detect using standard imaging techniques. The prevalence of hormone-active corticotroph adenomas is approximately 40 cases per million, with an annual incidence ranging from 1.1 to 2.3 cases per million. In adults, corticotroph adenomas are most-often diagnosed between the fourth and sixth decades of life and are approximately three times more prevalent in women. At the time of diagnosis, around 90% of patients present with corticotroph microadenomas measuring less than 1 cm in diameter. In some cases, these pituitary tumours are difficult to detect even with magnetic resonance imaging (MRI) [7,8,9].

Important progress has been made with the recent detection of recurrent gain-of-function mutations in the gene encoding a protein called ubiquitin-specific protease 8 (USP8) in about 20% to 60% of CAs in adult patients [10].

This study focused especially on the serum concentration of ACTH and cortisol post-surgery as predictive factors for the recurrence of the disease, and also on the surgical outcomes of the endoscopic transsphenoidal technique. In our study, no other pro-cancer factors or genetic mutations were studied.

The first-line treatment for patients with ACTH-secreting pituitary adenomas is surgical removal of the tumour, which typically results in the early normalisation of cortisol levels and symptom improvement [11,12,13].

In our study, it was essential to evaluate the initial outcomes of endoscopic surgery to determine its effectiveness and to identify potential intraoperative and postoperative complications.

It was also crucial to assess whether immediate postoperative ACTH and cortisol levels—measured in the blood and urine—serve as reliable markers of disease remission. While these biochemical parameters can provide early indications of treatment success, their long-term predictive value is limited. Given that disease recurrence may occur even years after surgery, determining the true relapse rate and identifying patients at risk of recurrence remain significant challenges.

## 2. Materials and Methods

Patients were enrolled from January 2013 to February 2023 in a retrospective cohort study at the University Hospital of Padova, Department of Neuroscience. After approval by its Ethics Committee, the medical records of patients who presented with Cushing’s disease were used. A total of 50 consecutive patients underwent resection of ACTH-secreting pituitary adenomas with the use of the endoscopic transsphenoidal approach. Demographic data are reported in Table 1.

### 2.1. Study Aims

This study aimed to evaluate the outcomes of endoscopic transsphenoidal surgery that might involve intraoperative and postoperative complications, as well as to evaluate the role of postoperative ACTH, serum cortisol, and urinary cortisol levels as predictors of long-term remission and delayed recurrence rates of Cushing’s disease.

In particular, by monitoring the hormonal markers in our patients, we tried to establish an association between early postoperative hypocortisolaemia and surgical success: in fact, it is believed that failure to achieve the timely suppression of morning serum cortisol levels in the postoperative period reflects the presence of disease residue, even at a microscopic level. We also assessed whether there is a correlation between certain factors such as gender, age, and tumour size.

### 2.2. Diagnosis

All patients underwent preoperative pituitary-specific triaxial contrast-enhanced three Tesla (3T) magnetic resonance imaging (MRI), including dynamic contrast scans of the pituitary gland. Each magnetic resonance image was blindly reassessed by an experienced neuroradiologist for the largest diameter of tumour, tumour location, and cavernous sinus involvement. Thirty-six (36) adenomas were classified as micro- and 14 were assessed as macroadenomas (Table 1 and Table 2). This study controlled for hypercortisolaemia, 24 h urinary free cortisol, dexamethasone suppression, and UFC in all patients. An ACTH measurement, CRH suppression test, desmopressin test, and high-dose dexamethasone test were then performed, which led to a diagnosis of ACTH-dependent Cushing’s disease. In 6 cases (12%), BIPSS was used prior to surgery to confirm the pituitary origin of the disease. The diagnosis of the syndrome was made on the basis of an endocrine society clinical practice guideline [14].

### 2.3. Previous Treatment

A total of 41 out of 50 patients had not undergone previous treatments. For the remaining 9, this was the second treatment, due to recurrent adenoma (7 patients had already undergone surgery and 2 had undergone radiotherapy). Obesity was highly prevalent (mean BMI 35.4 kg/m^2^) and insulin-dependent diabetes was reported in several cases (17.3%).

### 2.4. Preoperative Diagnosis of Cushing’s Disease

Cushing’s syndrome was confirmed on the basis of elevated results in at least two of the following laboratory tests: late-night salivary cortisol, 24 h urinary free cortisol, or low-dose-dexamethasone suppression testing (1 mg overnight or 2 mg over 48 h, with normal cortisol defined as <1.8 μg/dL). The ACTH level was also measured.

MRI was performed for all patients. In our cohort, two patients had inconclusive MRI findings with no clearly visible adenoma; therefore, IPSS was performed, confirming the diagnosis in both cases.

### 2.5. Surgical Treatment

**Figure 1:**
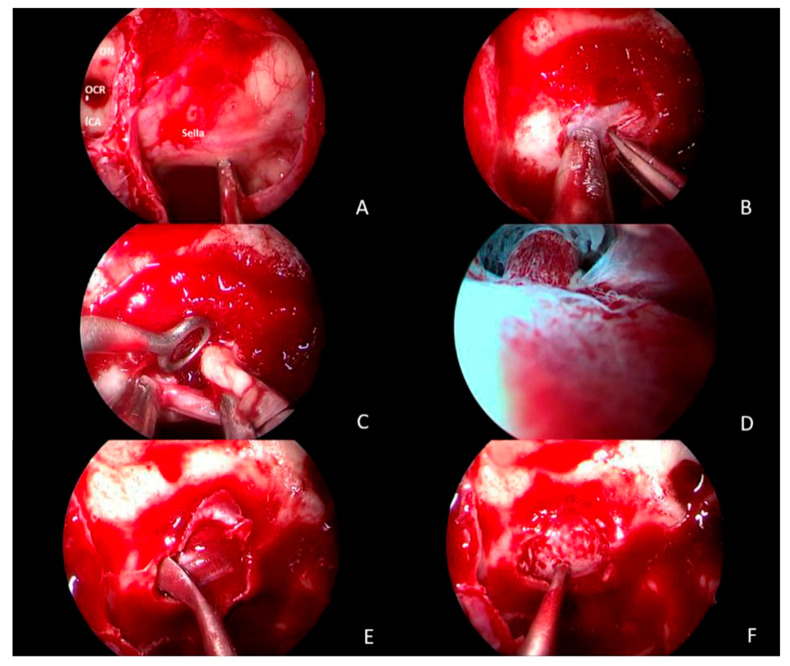
Endoscopic transsphenoidal approach. (**A**) View of sella turcica with opticocarotid recess (OCR), optic nerve (ON), internal carotid artery (ICA); (**B**) sella opening; (**C**) resection of the tumour with four-hand technique; (**D**) lavage and hydrodissection, with evidence of the pituitary stalk; (**E**,**F**) closure of the defect with gasket seal technique.

The TSS approach was performed in a total of 50 retrospective surgeries in the context of treatment of ACTH-secreting pituitary adenomas. An otolaryngologist and a neurosurgeon headed the surgical team in all operations. In no case was corticosteroid therapy administered during surgery (Figure 1).

### 2.6. Postoperative Management

Postoperatively, an evaluation was performed, which included the patients’ age at surgery and months of follow-up. Basal cortisol levels, and postoperative serum ACTH and UFC levels, which were defined as variables, were measured between 7:00 a.m. and 12:00 noon from day 1 to 10 and always at least 6 h after the administration of cortisone acetate, if prescribed.

Cortisol levels in the first 6 postoperative months were considered a prognostic factor. In the case of disease persistence after surgery, late hormonal data were not affected by the medical therapy of Cushing’s disease, which in no cases had yet been started. After the measurement of serum cortisol levels, on the third postoperative day, cortisone acetate therapy was initiated and continued at home (adjusted based on hormonal values).

### 2.7. Defining Postoperative Remission

Values between 270 and 500 nmol/L led to the decision that no treatment would be administered unless specific characteristics were identified in patients, such as a reoperation or the presence of stressful events or fever. Cortisol levels of >500 nmol/L (18 μg/dL), indicative of disease persistence, meant that patients should start medical therapy. A morning cortisol level of <5 μg/dL (<138 nmol/L) or urinary free cortisol (UFC) of <10–20 μg/24 h (<28–56 nmol/24 h) determined CD remission, as set out in the 2015 guidelines.

In all patients studied over time, we followed the criteria outlined in the relevant endocrinology guidelines, which defined the values used as prognostic factors. These were closely monitored throughout the study period.

None of the patients in this study underwent further surgery because, according to the surgeons, their tumours had been completely removed and, in one case, the tumour had eroded the cavernous sinus. None of the patients who relapsed underwent surgery. Re-operation was performed only when patients developed post-surgical complications such as a rhinoliquoral fistula.

The first postoperative MRI scan was performed four months after surgery, followed by annual scans in the absence of suspected disease recurrence.

### 2.8. Statistical Analysis

SPSS version 24.0 software was used to analyse the data. The Shapiro–Wilk normality test was used to study the distribution of the variables. The following variables were applied to compare patient subgroups: months of follow-up, postoperative serum cortisol and urinary cortisol, ACTH, BMI, and age. The application of the non-parametric Mann–Whitney U test yielded the comparison for the non-normal distribution of the variables.

## 3. Results

From January 2013 to February 2023, a total of 50 patients, comprising 34 females (69.7%) and 16 males (30.2%), with a median age of 37.8 years and a range of 15–69 years, underwent removal of an ACTH-secreting pituitary adenoma through the endoscopic endonasal approach. Median follow-up was 76.5 months (range: 23–122 months). After surgery, early remission was achieved in 84% of patients (n = 42/50). Eight (8) patients (16%) experienced disease persistence after surgery; of these, no patient underwent early re-intervention (Table 2).

For these eight patients, follow-up interventions were needed: one of them underwent a bilateral adrenalectomy due to poor control of the disease with medical therapy; another initially underwent conventional radiotherapy and subsequent medical therapy; and four more are still undergoing medical therapy with good control of their symptoms. In all cases, the adenoma had been identified intra-operatively and the surgeons had removed all of the visible tumour tissue.

Of the 42 patients who achieved early control, 39 remained in remission. Three patients with initial remission relapsed at 50, 60, and 62 months, respectively, after surgery. For all three patients, there were indications for surgical re-intervention. In two cases, a pituitary microadenoma was identified, with an early postoperative serum cortisol level of 120 nmol/L and 124 nmol/L, respectively. In a third case, a macroadenoma was diagnosed with an early postoperative serum cortisol level of 134 nmol/L by contrast-enhanced MRI. None of these three cases had previously undergone adenoma surgery. All three patients underwent surgery and are still disease-free.

### 3.1. Complications

Comparing patients with CD and those with non-functioning pituitary adenomas, the former may have an increased risk of postoperative complications. A transient CSF leak resulting from the surgical approach occurred in three patients. They were all treated with the application of an autologous graft with fascia lata. Neither recurrent CSF leak nor sequelae were seen in any patient. Two patients in the series developed a deep vein thrombosis and four more patients had a fever. In thirteen patients, transient diabetes insipidus (DI) occurred. Risk factors for the development of post-surgical DI are represented by tumour size, young age, and pathologies involving the neurohypophysis or the posterior portion of the pedicle. All complications are reported in Table 3.

In 9 out of 50 cases, the histological examination of the collected material did not reveal the presence of a pituitary adenoma. On preoperative imaging, a microadenoma was identified in all cases. All nine patients experienced disease remission following surgery and remain disease-free in the most recent follow-up.

### 3.2. Laboratory Analysis

Regarding the differences between males and females, the disease is more frequent in females; however, it tends to be more hormonally aggressive in males, as suggested by a significantly higher preoperative ACTH value found in the latter (*p* = 0.024), as shown in Table 4.

Moreover, by comparing the variable of tumour size (microadenomas and macroadenomas), we identified a difference, with higher values observed for macroadenomas (*p* = 0.07), although this was not statistically significant (Table 5).

However, a respective non-significance emerged when more variables were analysed between the subgroups. An immediate postoperative ACTH nadir of ≤5 pg/mL was significantly related to long-term remission (*p* = 0.004). It should be noted that the sample of macroadenomas is much smaller than that of microadenomas, which means that statistically, we cannot obtain reliable correlation results.

No statistically significant differences were found between the mean ACTH nadir and the last ACTH measurement. In both cases, the mean values were significantly lower in microadenomas, as well as for short- and long-term remission. Conversely, the mean values were higher in macroadenomas and in patients with the persistence and recurrence of CD after surgery.

In the first postoperative week, serum cortisol levels were evaluated at 8 a.m. daily. If hydrocortisone was prescribed, the measurement was made at least 6 h after taking it.

Early remission after surgery was achieved by 84% of patients, based on the cut-off value of 138 nmol/L. There was a significant difference (*p* < 0.001) between the remission group and that in which the disease persisted. Comparing these two groups, both the early serum cortisol level and the measurements made every three months after surgery for the first year of follow-up turned out to have statistically significant differences (*p* < 0.001), as shown in Table 6.

The cut-off value for early serum cortisol most often used in the literature is 138 nmol/L [15]. This defines the disease in remission. In our case, a high sensitivity of 100% and an acceptable specificity of 81% are noted.

The data relating to early and late postoperative serum cortisol levels were analysed by applying the ROC curve. Using the non-parametric Mann–Whitney U test, the acute value was found to be 0.087. A value of 135 nmol/L was derived; with a sensitivity of 100% and a specificity of 94%, it was the best cut-off value for early postoperative serum cortisol. Postoperatively, serum cortisol was found to be lower in men than in women. Although UFC, late measurement, did not vary significantly between males and females or between micro- and macroadenomas, it was significantly lower in patients in the remission group compared to patients in the persistence/non-remission group (*p* = 0.021), as shown in Table 3.

## 4. Discussion

A large cohort of patients with CD with prolonged follow-up was treated in a single centre by a surgical team consisting of the same members for the duration of the study. The surgical team specialises in advanced endoscopic and skull-base surgery and has gained significant experience over time, which has likely contributed to a reduction in complications. This study was initiated with an understanding of the rarity of the disease, aiming to identify beneficial insights into the postoperative management of these patients, and it was designed to report surgical outcomes and to investigate recurrence rates and potential contributing factors to recurrence. In this series of patients with CD, we demonstrated that TSS is safe and effective. Outcomes for both macro- and microadenomas are included, with an initial remission rate of 84% for those harbouring a microadenoma, with a median follow-up of 76.5 months (range: 23–122 months). Remission was achieved in 78% of cases (both macro- and microadenomas). Endoscopic endonasal transsphenoidal resection currently represents the gold standard of treatment, with remission rates ranging from 77% to 98% [16,17,18]. Immediate postoperative cortisol levels and long-term surgical success have been shown to be related. Moreover, as a predictive factor, low levels of ACTH of ≤5 pg/mL are associated with a low risk of recurrence [19,20,21].

### 4.1. Early Management Following Transsphenoidal Surgery for Cushing’s Disease

Transsphenoidal surgery is frequently challenging, and it is not unheard of for complications, sometimes serious, to arise. In all our patients, microadenomectomy was consistently performed as the first-choice procedure. There were three cases of CSF leak in our study, which were treated successfully, while pharmacotherapy was used for minor complications. Major complications reported in the literature occur at a varying rate, between 0% and 15%. Perioperative mortality ranges from 0% to 5% [13,14].

After surgery, 42 of the 50 patients in our study (84%) appeared to achieve remission, based on the cut-off value of 138 nmol/L. A statistically significant difference (*p* < 0.001) was calculated between the remission group and the persistence group regarding early serum cortisol. From the first week, low postoperative cortisol values had a high predictive value for remission of the disease. In terms of late serum cortisol, cortisol levels were stable for all patients at the 3-month follow-up. Despite this finding, no suitable value was found that would allow us to definitively rule out patients experiencing a relapse in the distant future [19].

### 4.2. The Role of Immediate Serum Cortisol and Urinary Cortisol

Even in our series, which has limited capabilities due to the low number of participants, an early postoperative serum cortisol level of <138 nmol/L manifested as patients having a distant recurrence of the disease. Of the 42 patients who achieved early control, 39 remained in remission but notably, not all of them belong to the patient group with an early postoperative serum cortisol level of <138 nmol/L.

The most sensitive method in use to deduce the presence of a persistent or relapsed tumour is believed to be the loss of the circadian rhythm of cortisol. Among several parameters that were taken into consideration in the follow-up, nocturnal salivary cortisol is the first to alter [22]. UFC is also recommended as an accurate measure for identifying Cushing’s disease. In our study, a significant correlation was confirmed between remission of the disease and 24 h urinary cortisol values, both early and late (*p* = 0.019).

In our series, 86.11% (n = 31/36) of microadenomas went into early remission after surgery and 80.55% (n = 29/36) into late remission. In larger sample studies, initial remission rates for microadenomas have ranged from 40 to 90%, with which our remission rates are comparable [23,24,25]. Our corresponding initial outcome of 78.57% (n = 11/14) for early remission of macroadenomas is also encouraging, since in other series it has been reported to amount to just 50% [18]. We are further encouraged by the fact that only 1/11 (9.1%) of those who had a resectable macroadenoma and initially went into remission later relapsed. No significant variation was recorded between males and females or between micro- and macroadenomas, compared to patients in whom the disease persists (*p* = 0.019), as shown in Table 4.

Some authors have argued in favour of an association between the histological confirmation of an ACTH-secreting adenoma and a positive prognostic value. In our series, despite the lack of histological confirmation in 24% of cases, all patients went into remission and were disease-free. Other authors have also reported lower remission rates, a finding on which we also converge, as a negative histology is inferred [26,27,28,29]; this has been recently confirmed in a large study [30,31].

### 4.3. Endocrine Assessment

In our study, the preoperative ACTH value and disease occurrence show no significant association. Some authors argue that if this value increases, it will reflect the size of the tumour rather than an increase in ACTH level [23]. An immediate postoperative ACTH nadir of ≤5 pg/mL was significantly related to long-term remission (*p* = 0.004). Although the available data are limited and contradictory, the dynamics of changes in plasma ACTH values after adenoma resection could also be of prognostic value. Patients who will subsequently achieve remission could be identified by an ACTH level reduction of >40% after the first hour post-surgery [32,33,34] or an early and rapid decrease in ACTH values [24,25,26,27,28]. Postoperative plasma ACTH levels were correlated with serum cortisol (r = 0.23; *p* < 0.001) and UFC (r = 0.29; *p* < 0.001) concentrations after surgery.

We calculated remission following the second surgery (nine patients), as we focused on the long-term outcome of this subgroup of patients. None of the nine patients relapsed during follow-up. The same finding has been reported by other authors in the literature [35,36,37,38,39,40,41,42].

Cushing’s disease, particularly in childhood, presents significant challenges in terms of both radiological diagnosis and clinical management [43]. According to the literature, the endoscopic transsphenoidal approach is indicated as a safe and effective treatment for paediatric CD, demonstrating high remission rates and minimal complications [43].

### 4.4. Remission

Finally, we have shown that the majority of patients with initial remission after TSS experienced substantial and sustained weight loss. Patients in postoperative remission had greater weight loss at 3 and 6 months relative to those without initial remission, outcomes, which are compatible with the literature [8].

Seven patients (16.6%) who did not exhibit an early postoperative cortisol level reduction subsequently achieved late remission (as aforementioned). This finding distinguishes our study from the prior literature. A gradual remission was detected in these patients, with early cortisol values of >138 nmol/L. Among them, a relapse occurred in one patient during follow-up. Specifically, in six patients, the immediate postoperative ACTH level was ≤5 pg/mL; in only one (who relapsed after ACTH), it was 5.8 pg/mL. We also saw that neither patient age nor size of the adenoma were related to the outcome.

### 4.5. Factors Influencing Remission

In the multivariable regression analysis, male gender was the only factor that was significantly associated with lower remission rates in either short- or long-term follow-up (*p* = 0.003 and 0.038, respectively). No significant relation was noted between the initial remission status and age (*p* = 0.17), gender (*p* = 0.74), and the duration of symptoms (*p* = 0.94). Moreover, postoperative plasma ACTH levels were correlated with serum cortisol levels.

Our study differs from the literature in that seven patients (16.6%) who did not exhibit an early postoperative cortisol level reduction subsequently achieved remission (also known as late remission, a phenomenon that, however rare, must be taken into account). We consider this to be an exceptional finding. Additionally, a gradual remission was observed in patients with early cortisol values of >138 nmol/L; then, at follow-up, one of these patients relapsed. In six patients, the immediate postoperative ACTH levels were ≤5 pg/mL. Only one was measured at 6.2 pg/mL and the patient then relapsed, manifesting ACTH.

Overall, although adrenal autonomy is an unlikely cause, the exact mechanisms behind the delayed decline in cortisol levels remain unclear and may involve factors such as the late necrosis of adenoma cells. Another extremely small possibility is that microscopic remnants of the pituitary adenoma subsequently died. The only patient who relapsed later than these patients had the characteristic of an elevated ACTH level immediately after surgery, which none of the other patients had. This may also be combined to gain some value as a predictive factor over time (Table 7).

ACTH levels have been used as a marker of remission in Cushing’s disease (CD). According to the literature, an ACTH level of less than 5 pg/mL postoperatively has a 100% positive predictive value for remission. Furthermore, cortisol and ACTH levels at all postoperative time points—including at 2 months—were found to be correlated. Our findings are consistent with those of previous studies [2,4].

### 4.6. Long-Term Follow-Up

Notably, three patients experienced recurrence of the disease after 4 and 5 years, without any preceding sign of disorder. No recurrence was seen in any patient in which HPA axis recovery occurred 3 years or more after the operation. The importance of continuing long-term assessment is emphasised by our finding of a 7.14% recurrence rate at 5 years, which is lower than in other studies that also claim that the rate increases to 20% at 10 years and to 24% at >10 years after surgery [4,33].

### 4.7. Strengths and Limitations

The small sample size, attributable to the rarity of ACTH-secreting pituitary adenomas, was a limitation in our study. Further validation of its accuracy from a multicentre prospective study with long-term follow-up would be a worthwhile endeavour. Considering the three relapses (7.14%) that occurred years later, any assessment of recurrence rates after surgical success in CD should include an extended duration of follow-up.

## 5. Conclusions

The involvement of a surgeon dedicated to the operative care of patients with pituitary disease is considered appropriate team reinforcement. This is reflected in the high success rates of the operation, with low proportional rates of relapses and complications. Our findings demonstrate that ACTH, postoperative serum cortisol, and urinary free cortisol levels are valuable predictors of relapse over a five-year period. Moreover, postoperative plasma ACTH levels are closely correlated with low levels of serum cortisol and urinary free cortisol. Additionally, in this study, we found that the risk of relapse in patients with CD persisting for more than 5 years after surgery is real but low (7.1% in our series), while 16.6% of patients who did not exhibit an early postoperative cortisol level reduction subsequently achieved late remission.

## Figures and Tables

**Table 1:** Demographic data.

Demographic Data	
Qualitative Variable	
**Gender**	
Female	34
Male	16
**Previous surgery in another centre**	
No	42
Yes	8
**MRI identification of the adenoma**	
Microadenoma	36
Macroadenoma	14
Diagnosis with IPSS	6
Previous treatment	9 (7 surgery, 2 radiotherapy)
First treatment	41
**Quantitative variable**	**Mean (S.D.)**
Age (years)	37.8 (11.02)
Pre-surgery UFC/ULC level (nmol/24 h)	239.08 (256.15)
Pre-surgery cortisol level (nmol/L)	429.09 (168.04)
Pre-surgery ACTH level (ng/mL)	74.25 (56)
BMI (kg/m^2^)	35.4 (8.5)
Insulin-dependent diabetes	17.3%
Follow-up	76.5 months

ACTH, adrenocorticotropic hormone; BMI, body mass index; IPSS, inferior petrosal sinus sampling; MRI, magnetic resonance imaging; UFC, urinary free cortisol; ULC, upper limit of normal level; SD, standard deviation.

**Table 2:** Biochemical data analysed by remission and non-remission of the patients.

Outcome of Surgery			
	Non-Remission	Remission	
N = 50	(N = 8)	(N = 42)	*p*-Value
**Age at surgery (years)**			
Median (IQR)	46.0 (40.0–53.0)	39.0 (26.0–47.0)	0.19
**Months of follow-up**			
Median (IQR)	58.0 (23.0–106.0	76.5 (23.0–122.0)	0.71
**ACTH, preoperative (ng/mL)**			
Median (IQR)	49.0 (38.0–100.0)	55.0 (44.0–102.0)	0.60
**Morning cortisol, preoperative (nmol/L)**			
Median (IQR)	487.5 (479.2–543.0)	438.0 (380.0–476.0)	0.12
**UFC, preoperative (nmol/24 h)**			
Median (IQR)	174.0 (128.0–430.9)	149.0 (109.0–248.0)	0.56
**Cortisol, postoperative (nmol/L)**			
Median (IQR)	449.5 (348.0–522.0)	57.6 (43.2–100.0)	<0.001
**UFC, early postoperative (nmol/24 h)**			
Median (IQR)	187.5 (71.0–319.0)	40.1 (26.5–99.0)	0.018
**ACTH, postoperative**			
Median (IQR)	42 (29.2–87.4)	5.1(4.2–13.4)	0.60

IQR, interquartile range (Q1–Q3); UFC, urinary free cortisol.

**Table 3:** Peri-operative and postoperative (30-day) surgical complications.

Demographic Data		
Qualitative Variable		
**Type of complication**	**Number of patients**	**Treatment**
	N = 50	50 surgeries
Cerebrospinal fluid rhinorrhoea	3	Autologous graft with fascia lata
Venous thromboembolic disease	2	Anticoagulant medicine
Fever	4	
Diabetes insipidus (DI)	13	
Hypothyroidism	5	Medical treatment
Anterior panhypopituitarism	2	Medical treatment
Anterior and posterior panhypopituitarism	3	Medical treatment
Growth hormone deficiency	3	Medical treatment

**Table 4:** Biochemical data analysed by gender.

	Gender		
	Female	Male	
N = 50	(N = 34)	(N = 16)	*p*-Value
**Age at surgery (years)**			
Median (IQR)	37.6 (33.0–49.0)	40.0 (29.0–59.0)	0.68
**Months of follow-up**			
Median (IQR)	76.5 (23.0–122.0)	76.4 (23.0–121.0)	0.16
**ACTH, preoperative (ng/mL)**			
Median (IQR)	49.5 (38.0–71.0)	109.0 (71.0–135.0)	0.024
**Morning cortisol, preoperative (nmol/L)**			
Median (IQR)	454.0 (389.0–485.0)	554.0 (459.0–611.0)	0.22
**UFC, preoperative (nmol/24 h)**			
Median (IQR)	139.0 (102.0–240.0)	218.0 (170.0–434.0)	0.23
**Cortisol, postoperative (nmol/L)**			
Median (IQR)	68.3 (44.0–321.0)	61.0 (33.0–129.0)	0.52
**UFC, postoperative (nmol/24 h)**			
Median (IQR)	71.0 (31.0–230.0)	34.0 (26.0–59.0)	0.11
**ACTH, postoperative**			
Median (IQR)	5.2 (4.1–13.4)	5.1 (4.4–12.7)	0.60

IQR, interquartile range (Q3–Q1); UFC, urinary free cortisol.

**Table 5:** MRI identification of the adenoma.

N = 50	Microadenoma (N = 36)	Microadenoma (N = 14)	*p*-Value
**Age at surgery (years)**			
Median (IQR)	38.0 (16.0–45.0)	50.0 (30.0–69.0)	0.13
**Months of follow-up**			
Median (IQR)	76.5 (23.0–122.0)	60.0 (25.0–121.0)	0.28
**ACTH, preoperative**			
Median (IQR)	48.0 (33.0–76.0)	85.3 (56.0–119.0)	0.09
**Morning cortisol, preoperative**			
Median (IQR)	469.0 (412.0–569.0)	454.0 (385.0–490.0)	0.65
**UFC, preoperative**			
Median (IQR)	141.0 (84.0–206.0)	238.0 (141.0–361.0)	0.25
**Cortisol, preoperative early**			
Median (IQR)	60.0 (43.0–222.0)	75.5 (57.0–320.0)	0.75
**Cortisol, preoperative late**			
Median (IQR)	28.0 (12.0–271.0)	97.0 (24.0–372.0)	0.27
**UFC, late**			
Median (IQR)	60.0 (32.0–227.0)	35.0 (13.0–72.0)	0.08
**ACTH, postoperative**			
Median (IQR)	5.1 (4.3–12.4)	5.3 (4.6–13.4)	0.09

IQR, interquartile range (Q1–Q3); UFC, urinary free cortisol.

**Table 6:** Examined serum cortisol, urinary cortisol, in remission and non-remission groups from the first week to three, six, and twelve months.

Outcome Measures	First Week	3 Months	6 Months	12 Months
**Serum cortisol (nmol/L)**				
Remission	95.81 (108.8)	95.70 (108.81)	95.22 (108.21)	91.55 (106.98)
Non-remission	471.16 (134.39)	470.66 (134.32)	466.83 (135.28)	462.83 (132.08)
**ACTH (pg/mL)**				
Remission	5.1 (4.2–13.4)	5.1 (4.1–13.2)	5.1 (4.1–13.3)	5.1 (4.2–13.6)
Non-remission	42 (29.2–87.4)	42 (29.3–87.8)	42 (29.6–88.1)	42 (29.7–88.4)
**UFC (nmol/24 h)**				
Remission	70.5 (71.66)	70.05 (70.76)	70.45 (70.86)	69.75 (71.22)
Non-remission	186.66 (121.68)	184.83 (120.63)	185.33 (121.25)	185.33 (122.32)

POD, postoperative day; UFC, urinary free cortisol.

**Table 7:** Characteristics of patients in early remission.

ID	Age	Sex	ACTH Pre-Surgery (ng/mL)	ACTH Post-Surgery (pg/mL)	UFC Pre-Surgery (nmol/24 h)	Serum Cortisol Pre-Surgery (nmol/L)	Previous Surgery	Size of Adenoma on MRI	UFC POD1 (nmol/24 h)	Serum Cortisol POD1 (nmol/L)	Serum Cortisol, 4-Month Follow-Up (nmol/L)	UFC Pre-Surgery (nmol/24 h)	Last Follow-Up Recurrence
1	35	F	65.30	4.9	138	390	No	Micro	94.3	134.39	97.32	36.0	No
2	42	F	56.29	6.2	141	368	No	Micro	110.9	160.45	110.23	87.74	YES
3	45	M	68.20	4.7	120	340	No	Micro	90.6	126.29	95.2	85.64	No
4	56	F	61.29	5.0	136	385	No	Micro	120.4	136.40	85.67	62.56	No
5	58	M	60.6	4.8	138	368	No	Micro	95.8	128.9	95.8	81.3	No
6	44	F	71.4	4.6	129	358	No	Macro	99.4	130.2	120.4	78.9	No
7	38	F	68.4	4.7	134	356	No	Micro	110.4	126.9	109.3	85.4	No

Micro, microadenoma; POD, postoperative day; UFC, urinary free cortisol.

## Data Availability

The data presented in this study are available on request from the corresponding author.
